# Unmasking Cardiac Sarcoidosis: Integrating Multimodal Imaging with Histochemical and Ultrastructural Analysis

**DOI:** 10.3390/ijms27072969

**Published:** 2026-03-25

**Authors:** Jakub Kancerek, Damian Świerczek, Wiktoria Baron, Marcin Rojek, Piotr Lewandowski, Romuald Wojnicz

**Affiliations:** Department of Histology and Cell Pathology, Faculty of Medical Sciences in Zabrze, Medical University of Silesia in Katowice, 41-808 Zabrze, Poland; s88516@365.sum.edu.pl (D.Ś.); s94372@365.sum.edu.pl (W.B.); marcin.rojek@365.sum.edu.pl (M.R.); lewandop@icloud.com (P.L.); rwojnicz@sum.edu.pl (R.W.)

**Keywords:** cardiac sarcoidosis, histochemistry, multimodal imaging, immunosuppression, cardiology

## Abstract

Cardiac sarcoidosis (CS) is a critical and frequently underdiagnosed phenotype of sarcoidosis, characterized by non-caseating granulomatous infiltration of the myocardium. This review synthesizes current knowledge regarding the pathogenesis, diagnosis, and management of CS. The disease manifests with a heterogeneous clinical spectrum ranging from asymptomatic conduction abnormalities to life-threatening ventricular arrhythmias and heart failure. Diagnosis remains challenging due to the patchy distribution of granulomas, which limits the sensitivity of endomyocardial biopsy. Consequently, a multimodal diagnostic approach is essential, integrating advanced imaging modalities such as cardiac magnetic resonance (CMR) with late gadolinium enhancement (LGE) and ^18^F-fluorodeoxyglucose positron emission tomography (FDG-PET). These tools not only facilitate detection but also enable the differentiation of active inflammation from chronic fibrosis. Histopathological assessment, supported by specific immunophenotyping and electron microscopy, remains the gold standard for confirming diagnosis and excluding mimics like giant cell myocarditis or infectious granulomatous diseases. Management requires a multidisciplinary strategy combining immunosuppressive therapy, primarily corticosteroids and steroid-sparing agents, with guideline-directed cardiac care, including implantable cardioverter-defibrillators for arrhythmia risk stratification. Emerging biomarkers and artificial intelligence-driven imaging analysis promise to further refine risk stratification and therapeutic monitoring, advancing precision medicine in this complex disorder.

## 1. Introduction

Sarcoidosis, historically known as Besnier–Boeck–Schaumann disease, is a multisystem granulomatous disorder of unknown aetiology defined by the histological presence of non-caseating, epithelioid, multinucleated giant-cell granulomas. While the condition manifests across all age groups and demographics, its epidemiological profile demonstrates significant variability based on geography and ethnicity. Prevalence estimates range widely from 2.17 to 160 per 100,000 persons, with notably higher incidence rates observed among Black and Hispanic populations in the United States, Caribbean residents, and Northern Europeans, particularly in Scandinavia. Although the median age at diagnosis is approximately 50 years, data indicates a bimodal distribution with incidence peaks at 25–29 and 65–69 years, where socioeconomic factors, sex, and ethnicity further influence disease expression and progression.

From a clinical assessment perspective, sarcoidosis is frequently termed a “clinical chameleon” due to its capacity to emulate a vast array of inflammatory, infectious, and neoplastic conditions, complicating the diagnostic process despite the disease’s substantial morbidity and mortality. The global burden of this condition is profound. According to recent estimates from the Global Burden of Disease (GBD) 2021 study, the broader category of sarcoidosis and interstitial lung diseases accounts for over 4.04 million disability-adjusted life years (DALYs) globally [1]. While epidemiological registries predominantly capture systemic and pulmonary data, cardiac sarcoidosis remains the leading cause of sarcoidosis-specific mortality. Consequently, undiagnosed or progressive cardiac involvement contributes disproportionately to these global DALYs, manifesting primarily as years of life lost (YLL) to sudden cardiac death and years lived with disability (YLD) due to progressive heart failure. While the pathology has the potential to infiltrate virtually any organ system, including the heart, liver, kidneys, eyes, and central nervous system, it exhibits a marked predilection for the thoracic cavity. Consequently, pulmonary involvement and intrathoracic lymphadenopathy constitute the predominant clinical phenotypes, accounting for over 90% of presentations [2,3,4]. The vast majority of cardiac diagnostics are performed as part of the systemic evaluation of patients with already established extracardiac sarcoidosis, most commonly involving the lungs. However, the demographic profile of patients developing cardiac sarcoidosis exhibits critical variations across sex, age, and race that must be recognized to prevent diagnostic bias. In the United States, while Black individuals are significantly more likely to develop systemic sarcoidosis (with an estimated incidence of 35.5 per 100,000 compared to 10.9 per 100,000 in White individuals), Black patients presenting with cardiac sarcoidosis are typically diagnosed at a younger age (mean age of 50.5 years versus 53.7 years for White patients) and present with lower baseline left ventricular ejection fractions [5,6]. Furthermore, pronounced sex disparities exist across populations. Studies indicate that females with cardiac sarcoidosis present at an older age than males (median age 58.5 versus 54 years) and frequently exhibit more severe symptomatic heart failure, including higher New York Heart Association (NYHA) functional classes at diagnosis [7]. Geographic phenotypes also heavily influence these demographic distributions, for example, in Japan, cardiac involvement is extraordinarily common, observed in 58% to 70% of autopsy cases and demonstrates a striking epidemiological skew towards females in their sixth decade of life [8]. In contrast, clinically isolated cardiac sarcoidosis, where granulomatous inflammation is strictly confined to the myocardium without any detectable systemic manifestations, is considered extremely rare, though it often poses a more significant diagnostic challenge and carries a poorer prognosis.

Cardiac sarcoidosis represents a critical and often underdiagnosed phenotype characterized by granulomatous infiltration of the myocardium and the cardiac conduction system. While clinically overt cardiac involvement is identified in only a small fraction of sarcoidosis patients, autopsy series suggest a significantly higher prevalence of subclinical disease, indicating that standard assessment methods frequently underestimate the burden of cardiac pathology. Because of its frequently asymptomatic or non-specific progression, a significant proportion of cardiac sarcoidosis cases remain undiagnosed during life and are ultimately identified post-mortem. Sudden cardiac death is often the first and only manifestation, underscoring the critical gap in early clinical detection.

## 2. Pathogenesis of Cardiac Sarcoidosis

The immunopathogenesis of cardiac sarcoidosis (CS) is postulated to originate from an aberrant, exaggerated T-cell-mediated immune response to unidentified antigens, potentially of microbial, environmental, or autoantigenic origin in genetically susceptible hosts. Current evidence suggests that specific human leukocyte antigen (HLA) class II alleles facilitate the presentation of these antigens to CD4+ T lymphocytes, triggering a breakdown in immune tolerance and the recruitment of mononuclear phagocytes to the myocardium. This inflammatory cascade is driven by a polarized Th1 cytokine profile, where the local release of interferon-gamma (IFN-γ), interleukin-2 (IL-2), interleukin-12 (IL-12), and tumour necrosis factor-alpha (TNF-α) orchestrates the aggregation of macrophages and their subsequent differentiation into epithelioid cells and multinucleated giant cells. These cellular aggregates form the non-caseating granuloma, the histological hallmark of the disease, which infiltrates the myocardial interstitium in a patchy, focal manner that does not respect coronary vascular territories [9,10]. At the molecular level, this cytokine-driven granulomatosis is heavily reliant on specific intracellular signaling cascades. Recent transcriptomic and mechanistic studies have identified hyperactivation of the mechanistic target of rapamycin (mTOR) complex 1 (mTORC1) in macrophages as a fundamental driver of their proliferation, aggregation, and metabolic reprogramming into epithelioid cells. Concurrently, the Janus kinase/signal transducers and activators of transcription (JAK/STAT) pathway plays a critical role in mediating the effects of IFN-γ and other Th1 cytokines. The activation of STAT1 and STAT3 pathways not only sustains the inflammatory milieu but also drives the expression of critical chemokines, notably the CXCL9/CXCL10/CXCL11–CXCR3 axis, which acts as a potent chemoattractant gradient for the continuous recruitment of peripheral CD4+ T cells into the myocardial interstitium [11].

**Figure 1:**
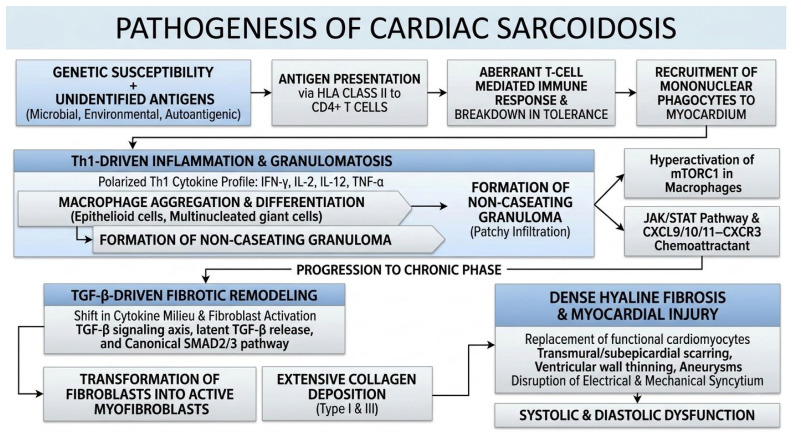
Pathogenesis of cardiac sarcoidosis.

Crucially, the disease process is not static; the granulomatous inflammation frequently progresses to a chronic phase characterized by extensive fibrotic remodelling. While the mechanisms governing this transition remain partially defined, it involves a shift in the local cytokine milieu that promotes fibroblast activation and collagen deposition, resulting in the replacement of functional cardiomyocytes with dense, hyaline fibrosis. In the heart, this scarring is often transmural or subepicardial and leads to significant architectural distortion, including thinning of the ventricular walls and the formation of aneurysms [9,12]. At a molecular level, this fibrotic transition is primarily orchestrated by the Transforming Growth Factor-beta (TGF-β) signaling axis. The persistent inflammatory state and localized tissue injury trigger the release of latent TGF-β, which binds to its cell-surface receptors and activates the canonical SMAD2/3 signaling pathway. The nuclear translocation of SMAD complexes profoundly alters the transcriptional profile of resident cardiac fibroblasts, driving their transdifferentiation into highly active myofibroblasts. This results in the overwhelming local synthesis of extracellular matrix proteins, predominantly type I and type III collagens, fundamentally disrupting the electrical and mechanical syncytium of the heart [11]. This irreversible loss of contractile tissue contributes directly to systolic and diastolic dysfunction, distinguishing CS from other cardiomyopathies by its unique pattern of non-ischemic, patchy myocardial injury (Figure 1) [9,12].

## 3. Clinical Presentation of Cardiac Sarcoidosis

The clinical spectrum of CS is notably heterogeneous, ranging from asymptomatic conduction abnormalities to life-threatening ventricular arrhythmias, high-grade heart block, and progressive heart failure. As cardiac involvement constitutes a leading cause of sarcoidosis-related mortality, early identification is paramount, though diagnosis remains challenging due to the focal and patchy nature of myocardial inflammation, which often evades histological detection [13,14].

The mechanistic basis for the life-threatening arrhythmias associated with cardiac sarcoidosis lies in this heterogeneous admixture of active granulomas, fibrotic scar, and viable myocardium. The infiltration typically exhibits a marked predilection for the basal interventricular septum, directly compromising the atrioventricular node and the His-Purkinje system, which explains the high prevalence of atrioventricular block as a presenting sign. Furthermore, the interspersed fibrosis creates distinct zones of slow conduction and unidirectional block, forming the anatomical substrate necessary for macro-reentrant circuits. These circuits are the primary drivers of sustained ventricular tachycardia and sudden cardiac death, occurring even in patients with preserved left ventricular ejection fraction, thereby underscoring the critical disconnect between gross mechanical function and electrical instability in this pathology [12,15].

## 4. Non-Invasive Diagnosis

Non-invasive diagnostic evaluation plays a central role in the detection and characterization of cardiac sarcoidosis, particularly given the limited sensitivity of endomyocardial biopsy due to the patchy distribution of myocardial involvement.

Transthoracic echocardiography (TTE) is often the first-line non-invasive modality used in the evaluation of suspected cardiac sarcoidosis, owing to its wide availability and ability to assess cardiac structure and function. Although no single echocardiographic feature is pathognomonic for sarcoidosis, characteristic findings may include regional wall motion abnormalities (not in a coronary distribution), basal septal thinning, ventricular aneurysms, left- or right-ventricular systolic or diastolic dysfunction, and chamber dilatation. However, it is crucial to recognize that the overall sensitivity of standard TTE for detecting cardiac sarcoidosis is notably poor, as it frequently fails to identify early, localized, or subclinical granulomatous infiltration [16]. More sensitive detection of subclinical myocardial involvement can be achieved through speckle-tracking echocardiography (STE): meta-analyses show that patients with extracardiac sarcoidosis have significantly reduced global longitudinal strain (GLS) despite preserved ejection fraction, indicating early contractile impairment [17]. In a biopsy-proven sarcoidosis cohort, segmental strain abnormalities on STE correlated with late gadolinium enhancement on cardiac MRI, suggesting its potential utility as a screening and monitoring tool [18].

**Figure 2:**
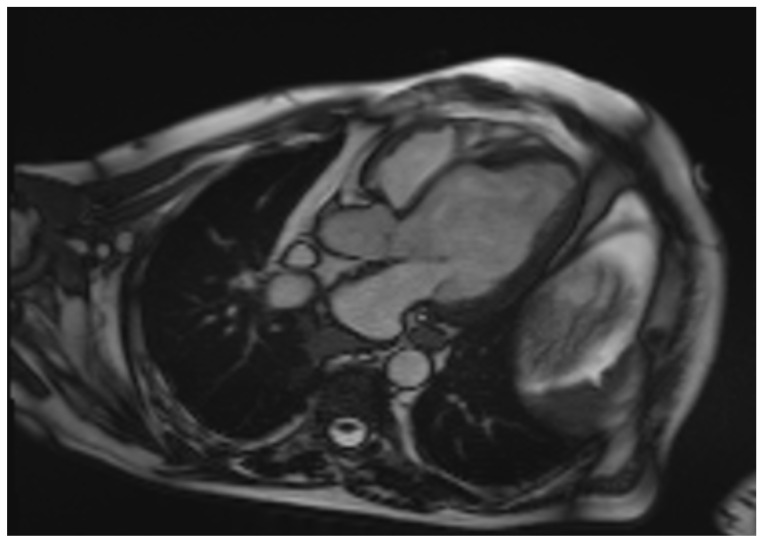
Cardiac Magnetic Resonance (CMR) four-chamber view demonstrating severe structural remodeling. This image reveals significant biventricular dilatation and pronounced, pathological thinning of the interventricular septum. Such gross architectural distortion, often accompanied by regional wall motion abnormalities, septal dyskinesia, and a severely reduced left ventricular ejection fraction is highly characteristic of advanced cardiac sarcoidosis. These structural changes reflect the extensive replacement of healthy contractile myocardium with metabolically inactive, dense fibrotic scar tissue.

**Figure 3:**
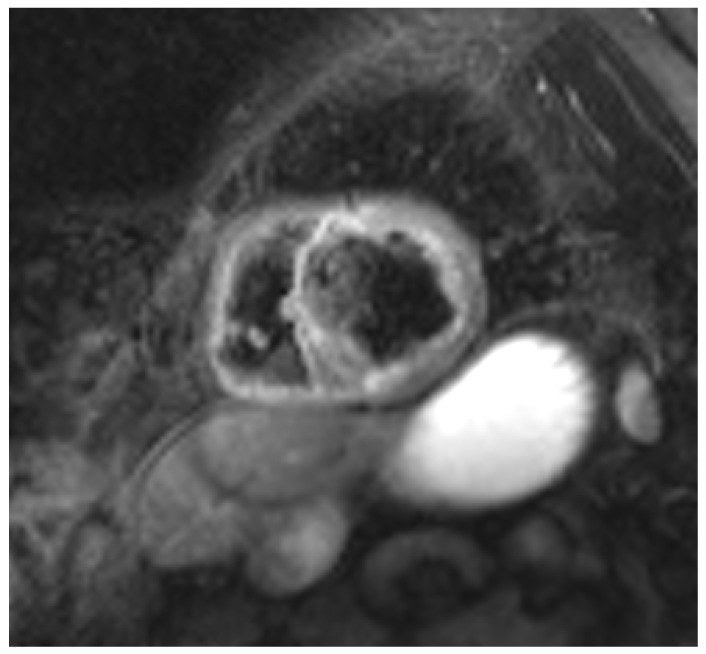
Cardiac Magnetic Resonance (CMR) short-axis view demonstrating focal myocardial injury and active inflammation. This cross-sectional view highlights prominent areas of hyperintensity localized predominantly within the anteroseptal and anterior left ventricular walls. In the context of active cardiac sarcoidosis, these elevated signals correlate directly with the localized myocardial edema and transmural scarring detailed in clinical evaluations. This patchy, non-ischemic pattern of myocardial involvement is a hallmark imaging signature of the disease, visualizing the anatomical substrate responsible for both severe heart failure and life-threatening ventricular arrhythmias.

Cardiac magnetic resonance (CMR) (Figure 2 and Figure 3) is a pivotal non-invasive imaging modality for the evaluation of suspected cardiac sarcoidosis, offering high spatial resolution and tissue characterization. The hallmark CMR finding is late gadolinium enhancement (LGE), which indicates myocardial fibrosis or inflammation. In sarcoidosis, LGE typically presents in non-ischemic patterns such as sub-epicardial or mid-myocardial enhancement of the basal septum or inferolateral wall, often sparing the subendocardium [19]. Regions of myocardial edema may also be detected on T2-weighted or T2-mapping sequences, reflecting active inflammation. Importantly, the presence of LGE on CMR has strong prognostic significance. Meta-analyses demonstrate that patients with LGE have substantially higher risks of ventricular arrhythmias and all-cause mortality [20]. Furthermore, the specific phenotypic pattern of myocardial involvement visualized via CMR provides critical prognostic insights; the precise topographic distribution and burden of LGE correlate strongly with distinct clinical trajectories and arrhythmic risk, highlighting CMR’s indispensable role in comprehensive disease phenotyping [21].

FDG-PET is a key non-invasive modality for assessing active myocardial inflammation in cardiac sarcoidosis and serves as an important complement to CMR. Meta-analyses including 1363 patients demonstrate pooled sensitivity and specificity of approximately 81% and 82%, respectively, with sensitivity increasing to 93% when quantitative analysis of FDG uptake is applied rather than qualitative interpretation [22,23]. A recent comprehensive systematic review and meta-analysis of FDG PET/CT further corroborates these findings, affirming its robust diagnostic accuracy and underscoring its superior utility in differentiating active inflammatory phases from chronic scarring [24]. FDG-PET detects inflammation by exploiting the increased glucose consumption of activated macrophages and T lymphocytes, which take up F-18 labelled glucose via GLUT transporters, leading to intracellular trapping after phosphorylation by hexokinase. Typical findings include multifocal regions of increased FDG uptake, often accompanied by perfusion defects and extracardiac inflammatory activity [22]. Proper metabolic preparation, such as a high-fat, low-carbohydrate diet, prolonged fasting, and, in some cases, heparin administration, is essential to suppress physiological myocardial glucose uptake and enhance lesion visualization [25]. Quantitative PET metrics, particularly serial SUVmax measurements, provide prognostic information and enable monitoring of therapeutic response during immunosuppressive treatment [26].

**Figure 4:**
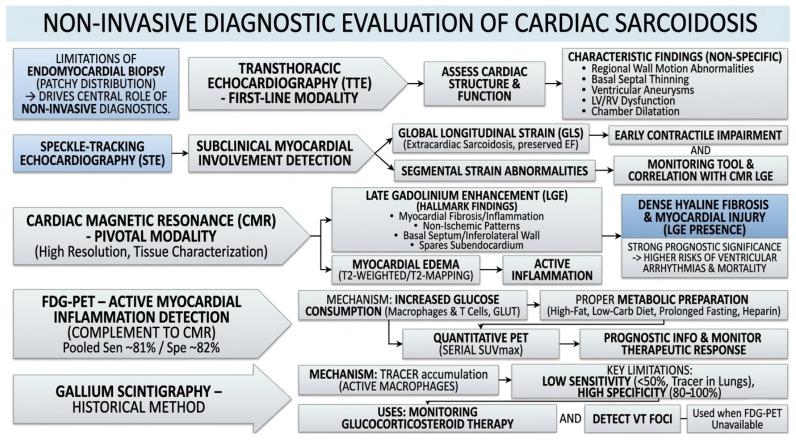
Non-invasive diagnostics of cardiac sarcoidosis.

Gallium scintigraphy is one of the oldest methods of imaging inflammation used in cardiac sarcoidosis. This method shows a relatively high specificity of 80–100%, but very low sensitivity of less than 50% due to possible tracer uptake by mediastinal and pulmonary structures. The mechanism of gallium scintigraphy involves tracer accumulation within metabolically active macrophages at sarcoid lesion sites, allowing assessment of tracer accumulation and the density of granulomatous lesions in the heart [27]. Gallium scintigraphy has historically been used primarily to monitor glucocorticosteroid therapy in patients with cardiac sarcoidosis. Due to its ability to image localized regions, it may also be used to detect ventricular tachycardia caused by sarcoid granuloma accumulation in cardiac structures (Figure 4) [27]. Due to its low sensitivity, gallium scintigraphy is less commonly used than FDG-PET, which has a higher sensitivity of 81% and better contrast resolution [25]. For this reason, scintigraphy is currently used mainly where FDG-PET is unavailable [27].

## 5. Biopsy-Based Diagnosis

Endomyocardial biopsy is a key tool for histopathological confirmation of cardiac sarcoidosis, and when combined with polymerase chain reaction (detecting pathogenic microorganisms) and immunohistochemistry (analysing the cellular composition of inflammatory infiltrates), it constitutes the diagnostic gold standard for sarcoidosis [28,29]. In a study of 26 patients with cardiac sarcoidosis, non-caseating granulomas were found in 5 patients, and sarcoid lesions were identified in only 9 of 105 biopsy samples [30]. Due to the localised and uneven distribution of focal lesions in cardiac sarcoidosis, its diagnostic accuracy is approximately 20%. Therefore, if abnormalities are detected in the heart using other diagnostic methods, treatment should be initiated despite a negative biopsy result [31]. The diagnostic effectiveness of endomyocardial biopsy can be improved by simultaneous use of ECG and specific imaging methods, such as cardiac magnetic resonance [29]. Furthermore, to overcome the limitations of blind sampling, electroanatomical mapping-guided endomyocardial biopsy is increasingly being utilized. By targeting specific areas of low electrical voltage or fragmented electrograms, which correspond to fibrotic or inflamed myocardium, this technique significantly improves the diagnostic yield and helps precisely localize the patchy granulomatous infiltrates [32]. Despite its low sensitivity, the method is used to distinguish cardiac sarcoidosis from giant cell myocarditis, which is important for treatment planning and determining patient survival [33].

A typical feature of the histopathological presentation of sarcoidosis is the presence of granulomas, usually lacking necrosis. Granulomas are composed of dendritic cells, monocytes, activated macrophages resembling squamous epithelial cells, T helper cells facilitating granuloma formation through cytokine secretion, cytotoxic T cells, Langerhans-type giant cells, and small numbers of plasma cells contributing to humoral immunity [34]. After the active inflammatory phase, granulomatous lesions are replaced by metabolically inactive fibrous tissue. Extensive myocardial fibrosis may lead to conduction abnormalities and heart failure [22]. In the kidney, granulomas are usually confined to the cortex, where they cause interstitial fibrosis, tubular atrophy, and formation of single calcifications [35].

## 6. Histochemistry Typing

**Figure 5:**
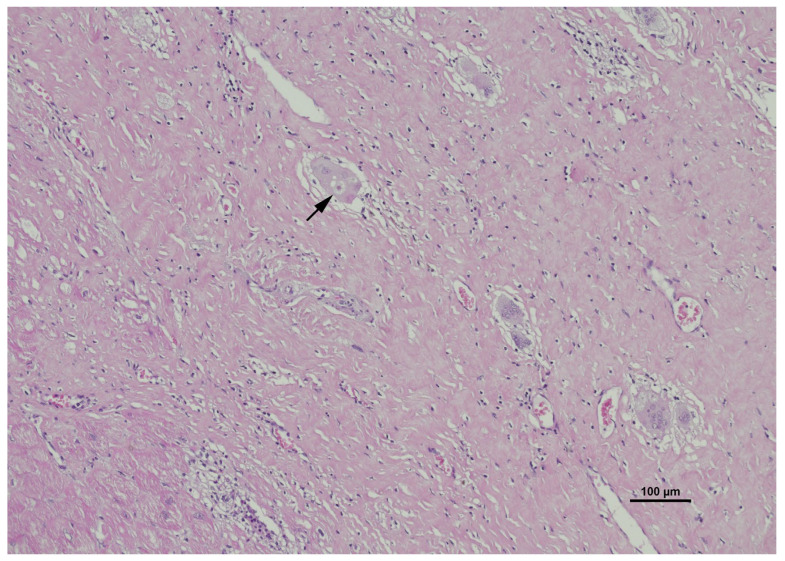
Hematoxylin and Eosin (H&E) staining of myocardial tissue demonstrating a characteristic asteroid body. The black arrow highlights a distinct, star-shaped intracytoplasmic inclusion known as an asteroid body or stellate inclusion housed within a large multinucleated giant cell. These eosinophilic structures are frequently observed within granulomatous infiltrates on standard light microscopy. While not exclusively pathognomonic, the presence of such prominent stellate formations amidst the disrupted, fibrotic myocardial architecture strongly supports the morphological diagnosis of cardiac sarcoidosis.

The histopathologic analysis of cardiac sarcoidosis is the presence of discrete, non-caseating epithelioid cell granulomas within the myocardial tissue [36,37]. These granulomatous lesions are architecturally distinct, characterized by a compact core of activated macrophages that have differentiated into epithelioid histiocytes, frequently accompanied by multinucleated giant cells of the Langhans or foreign-body type [36]. Within these multinucleated giant cells, characteristic intracytoplasmic inclusions can frequently be observed on standard light microscopy. These include prominent star-shaped, eosinophilic structures known as asteroid bodies (stellate inclusions), as well as laminated basophilic calcifications called Schaumann bodies (Figure 5). While not exclusively pathognomonic, their presence strongly supports the morphological diagnosis of sarcoidosis. In contrast to the necrotizing granulomas observed in tuberculous myocarditis, sarcoid granulomas typically preserve cellular integrity at their centres, lacking the amorphous caseous necrosis that defines mycobacterial infections [36,38]. Over the temporal course of the disease, this active granulomatous phase often evolves into a chronic fibrotic stage. The persistent inflammation triggers fibroblast activation, leading to dense hyaline fibrosis and scar formation [37,39]. This replacement of functional myocardium with fibrotic tissue explains the gross morphological sequelae often observed, such as myocardial wall thinning and the development of ventricular aneurysms [40].

The topographic distribution of these lesions within the heart is characteristically heterogeneous and focal, a feature that significantly complicates diagnostic sensitivity [38,41]. The granulomas exhibit a marked predilection for specific anatomical sites, most notably the basal interventricular septum, the left ventricular free wall, and the papillary muscles [42]. This “patchy” dissemination serves as the primary limitation of endomyocardial biopsy (EMB), while the histological identification of non-caseating granulomas remains the diagnostic gold standard, the focal nature of the disease results in substantial sampling error, meaning a negative biopsy cannot definitively exclude cardiac sarcoidosis in the setting of high clinical suspicion [38,41]. Furthermore, the concept of isolated cardiac sarcoidosis, where granulomatous inflammation is confined solely to the heart without systemic involvement, underscores the critical need for meticulous histopathological evaluation of myocardial tissue when clinical presentation suggests the disease [41].

**Figure 6:**
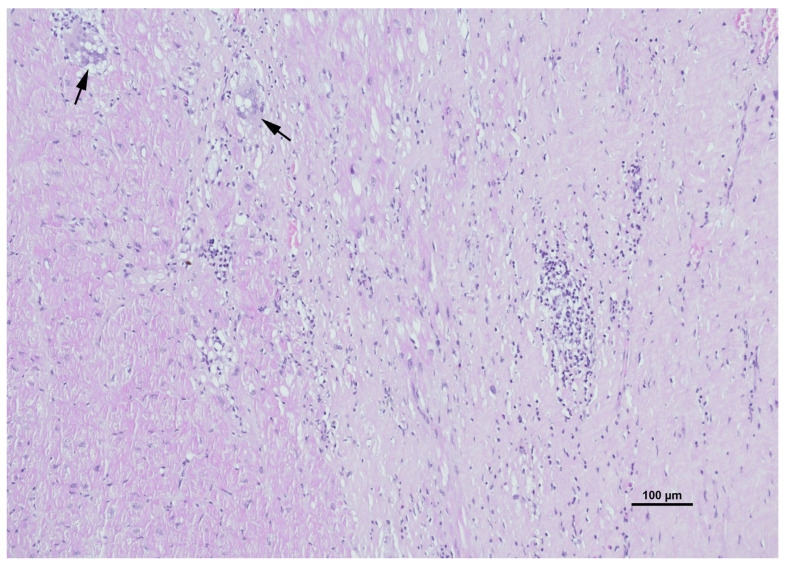
Hematoxylin and Eosin (H&E) staining of myocardial tissue demonstrating granulomatous inflammation. The black arrows highlight multinucleated giant cells, a hallmark of the granulomatous process, embedded within a background of inflammatory infiltrate. Surrounding these giant cells are clusters of epithelioid histiocytes and lymphocytes disrupting the normal myocardial architecture, consistent with the non-caseating granulomas characteristic of cardiac sarcoidosis.

**Figure 7:**
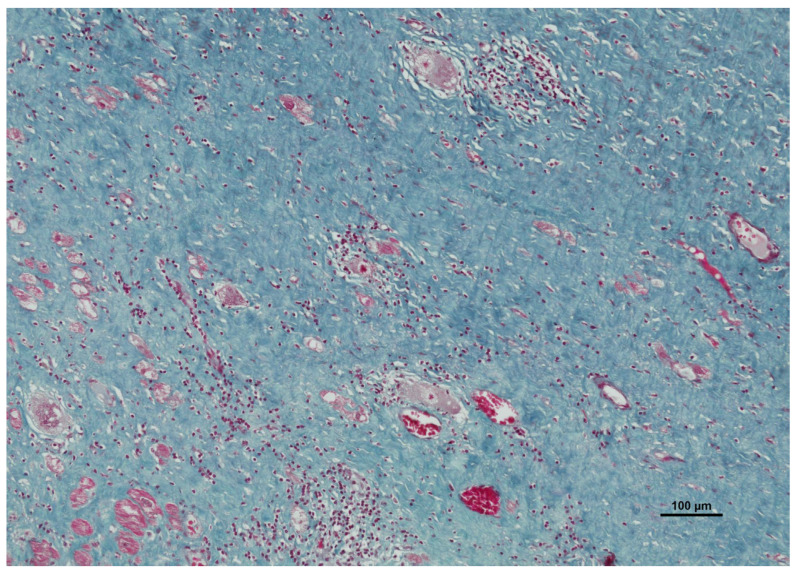
Masson’s trichrome staining of myocardial tissue revealing extensive fibrosis. In this stain, collagen fibers are highlighted in blue, indicating dense interstitial and replacement fibrosis that has superseded active inflammation. The scattered islands of surviving cardiomyocytes appear red, appearing entrapped and fragmented within the fibrotic matrix. This pattern reflects the chronic stage of the disease, where persistent granulomatous inflammation leads to permanent scarring and structural remodeling of the myocardium.

Given that the morphological appearance of sarcoid granulomas can overlap significantly with infectious granulomatous myocarditis, the diagnosis is fundamentally one of exclusion, necessitating a rigorous histochemical staining protocol (Table 1) [36,43]. Routine hematoxylin and eosin (H&E) (Figure 6) staining is utilized for the primary assessment of tissue architecture, allowing for the visualization of the granulomatous infiltrates, giant cells, and associated fibrosis [37]. Complementing this, Masson’s trichrome staining (Figure 7) is frequently employed to highlight collagen deposition, distinguishing fibrous replacement from active inflammation and providing a clearer assessment of the chronicity and extent of myocardial scarring.

However, to rule out infectious etiologies, specific special stains are mandatory [36]. The Ziehl–Neelsen (ZN) acid-fast stain is applied to detect the presence of acid-fast bacilli, thereby excluding Mycobacterium tuberculosis and atypical mycobacteria [36,38]. Concurrently, Periodic acid-Schiff (PAS) and Grocott’s methenamine silver (GMS) stains are employed to visualize fungal cell walls and polysaccharide-rich structures, excluding fungal pathogens such as Histoplasma or Aspergillus [43]. A diagnosis of cardiac sarcoidosis is histopathologically supported only when these special stains yield negative results demonstrating an absence of detectable acid-fast organisms or fungal elements in the presence of the characteristic non-necrotizing granulomatous inflammation [36,44].

## 7. Immunohistochemistry Typing

In the granulomatous lesions of cardiac sarcoidosis, immunohistochemical analysis typically reveals a pattern characterised by macrophages positive for CD68, interspersed with T lymphocytes expressing CD3, and with a predominance of CD4+ helper T cells over CD8+ cytotoxic T cells. CD3 immunostaining strongly highlights T cells, while CD4 antibody staining intensely marks T cells in myocardial granulomas and, to a lesser extent, macrophages and giant cells. The macrophage/giant cell compartment is readily identifiable by CD68 immunostaining. This immunophenotypic profile reflects a T helper cell-macrophage driven granulomatous immune response in the myocardium, suggesting that the predominance of CD4+ T cells may be indicative of the immune dysregulation inherent to sarcoidosis rather than a purely cytotoxic or infectious process [37].

Complementing cellular immunophenotyping, inflammatory biomarkers provide valuable adjunctive information for assessing disease activity. Angiotensin-converting enzyme (ACE), soluble interleukin-2 receptor (sIL-2R), and tumour necrosis factor-α (TNF-α) have all been implicated in the pathophysiology of sarcoid granulomas. ACE is produced by epithelioid and giant cells within granulomas, and elevated serum levels reflect granuloma burden, although sensitivity is modest and influenced by genetic polymorphisms and ACE-inhibitor therapy [45]. sIL-2R, released by activated T lymphocytes, serves as a sensitive measure of T-cell activation. In one large study, it outperformed ACE in discriminating sarcoidosis from other conditions (sensitivity 88%, specificity 85% versus 62% and 76%, respectively) [46]. TNF-α, a cytokine central to granuloma formation and maintenance, is elevated in active disease compared with stable sarcoidosis, highlighting its potential role as a marker of disease activity and prognosis [47]. While these biomarkers are not entirely specific for cardiac sarcoidosis, their integration with imaging and histologic data strengthens the assessment of active myocardial inflammation and informs therapeutic decision-making.

Beyond diagnosis and activity assessment, prognostic markers have emerged to identify patients at higher risk of adverse outcomes. Elevated expression of HLA-DR on macrophages within granulomatous lesions identifies subpopulations with high antigen-presenting capacity and is associated with more aggressive or persistent inflammation [48]. In parallel, myocardial fibrosis (fibrogenesis) is increasingly recognised as a key determinant of clinical outcomes. Surrogate markers such as circulating fibrocytes (CD34^+^/collagen-1^+^) and endothelial progenitor cell dysregulation correlate with fibrotic progression in sarcoidosis, and the presence of confluent fibrosis and fatty infiltration on endomyocardial biopsy, even in the absence of overt granulomas, has been linked to a “probable” diagnosis of cardiac sarcoidosis and worse prognosis [49,50]. Taken together, the combination of HLA-DR expression and a fibrotic myocardial substrate offers a promising approach for risk stratification, potentially guiding earlier or more intensive therapy to prevent severe complications such as arrhythmias or heart failure.

## 8. Electron Microscopy

Electron microscopy (EM) serves as a high-resolution adjunctive modality in the investigation of cardiac sarcoidosis, offering critical insights into the ultrastructural architecture of granulomatous inflammation that exceed the capabilities of light microscopy. While routine diagnosis relies on histology, EM reveals the complex cellular interactions driving granuloma formation, specifically visualizing the tight interdigitation of epithelioid histiocytes and the fusion events between monocytes and multinucleated giant cells. Ultrastructural analysis captures the “fusogenic” potential of macrophages, documenting the close apposition of cell membranes and cytoplasmic continuity that precede Giant Cell formation, a process central to the pathogenesis of the disease [51,52]. Furthermore, EM permits the detailed characterization of cytoplasmic inclusions often found within these giant cells, such as laminated calcific Schaumann bodies and stellate asteroid bodies, distinguishing them from foreign material or other cellular debris [53]. Beyond characterizing the granuloma itself, EM is invaluable in the exclusion of specific mimics. It allows for the definitive ultrastructural search for viral particles or mycobacterial fragments, thereby helping to differentiate sarcoidosis from infectious myocarditis or Giant Cell Myocarditis when conventional stains are equivocal [54]. Although technical complexity limits its routine clinical use, EM remains a powerful tool for elucidating the immunopathogenesis of cardiac sarcoidosis and resolving diagnostically difficult cases.

While conventional electron microscopy provides invaluable ultrastructural details, the required chemical fixation, dehydration, and resin embedding processes inherently risk introducing structural artifacts that can alter the native state of lipid membranes and protein complexes. Consequently, the application of cryogenic electron microscopy (cryo-EM) and correlative multi-scale cryo-imaging represents a highly promising frontier for preserving true sample integrity. By rapidly freezing and vitrifying the specimens without the use of chemical fixatives, these advanced cryo-imaging techniques maintain the native, hydrated state of the cellular architecture, as has been successfully demonstrated in complex viral and cellular models. In the context of cardiac sarcoidosis, adapting this methodology could allow for the remarkable, artifact-free visualization of macrophage fusion dynamics and the precise macromolecular organization of intracytoplasmic inclusions, such as asteroid bodies. Although the extreme technical demands currently limit its routine diagnostic use, its future integration into basic research holds immense potential for uncovering the unaltered molecular pathogenesis of sarcoid granulomas [55].

## 9. Differential Diagnosis

The differential diagnosis of Cardiac Sarcoidosis (CS) is challenging due to overlapping clinical, imaging, and histopathological features with other myocardial diseases. Emerging diagnostic tools help improve discrimination by integrating functional, structural, and molecular data. The spectrum of conditions mimicking CS includes infiltrative, inflammatory, and infectious myocardial diseases. Cardiac amyloidosis is characterised by diffuse amyloid-fibril deposition and concentric left-ventricular hypertrophy, whereas CS more often exhibits patchy myocardial involvement with conduction disturbances and ventricular arrhythmias [56]. Hypertrophic Cardiomyopathy (HCM) may present with asymmetric septal hypertrophy; however, in CS, CMR typically reveals sub-epicardial or mid-myocardial Late Gadolinium Enhancement (LGE), often in the basal septum, which is uncommon in HCM [57]. The diagnostic accuracy of these advanced imaging modalities is paramount. Recognizing the highly specific LGE and FDG uptake patterns allows clinicians to reliably differentiate cardiac sarcoidosis from an array of complex phenocopies and mimics, thereby preventing misdiagnosis and guiding appropriate, disease-specific therapies [58].

Myocarditis, including viral or lymphocytic forms and eosinophilic myocarditis, can mimic CS with arrhythmias or heart failure, but these conditions generally show diffuse inflammatory infiltrates or eosinophil-rich histology rather than the non-caseating granulomas characteristic of sarcoidosis [59]. Infectious granulomatous myocarditis, caused by tuberculosis or fungal pathogens, may resemble CS on imaging but typically presents with caseating necrosis or identifiable organisms on special stains or molecular testing [60].

Given the overlap in clinical and imaging features among these entities, a robust diagnosis of cardiac sarcoidosis requires careful correlation of clinical, imaging, and pathological findings. Clinically, patients may present with conduction disturbances, arrhythmias, or heart failure features shared with many mimicking conditions. Correlating advanced imaging patterns with histologic features significantly enhances diagnostic confidence [61]. As emphasized in the literature, successful recognition of CS requires close collaboration among clinicians, radiologists, and pathologists, with integrated imaging-pathology correlation improving diagnostic accuracy beyond either modality alone [62]. This clinical-image-pathology triad remains fundamental not only in distinguishing CS from its mimics but also in guiding immunosuppressive therapy versus alternative management strategies.

## 10. Treatment and Monitoring

Management of cardiac sarcoidosis requires a multidisciplinary approach that simultaneously addresses myocardial inflammation, arrhythmias, and heart failure. Immunosuppressive therapy remains the cornerstone of treatment, aiming to control granulomatous inflammation and prevent progression to myocardial fibrosis. Corticosteroids, particularly prednisone, are first-line agents, typically initiated at moderate to high doses based on disease severity and tapered according to clinical and imaging response [22,63]. In patients with incomplete response, steroid intolerance, or a high cumulative steroid burden, additional immunosuppressive agents are employed: methotrexate is commonly used as a steroid-sparing agent, azathioprine serves as an alternative, and infliximab, a TNF-α inhibitor, is reserved for refractory cases targeting persistent granulomatous inflammation [22,64]. Treatment selection is guided by the extent of myocardial involvement, disease activity, comorbidities, and the risk of adverse effects, with regular imaging and clinical follow-up to monitor efficacy.

Alongside immunosuppressive therapy, cardiologic management is essential to reduce the risk of arrhythmias and heart failure. Patients at high risk for ventricular tachyarrhythmias or sudden cardiac death may benefit from an implantable cardioverter-defibrillator (ICD), particularly those with prior sustained ventricular arrhythmias, severely reduced left ventricular ejection fraction, or significant conduction disease. Standard heart failure therapies, including beta-blockers, ACE inhibitors or ARBs, mineralocorticoid receptor antagonists, and diuretics, are applied as indicated. Coordinating immunosuppressive therapy with cardiologic interventions allows simultaneous control of inflammation and stabilization of cardiac function, improving overall outcomes [64,65].

Assessment of treatment response increasingly relies on advanced imaging modalities. FDG-PET provides a sensitive measure of active myocardial inflammation, with reductions in focal uptake indicating effective suppression of granulomatous activity. Cardiac MRI (CMR) complements PET by evaluating structural changes, including edema resolution and monitoring of late gadolinium enhancement (LGE) to track fibrosis progression or regression. Sequential imaging enables clinicians to adjust the intensity of corticosteroid or adjunctive immunosuppressive therapy, identify persistent active disease, and optimize timing of interventions. Studies demonstrate that patients with decreased FDG uptake and stable or reduced LGE on follow-up exhibit improved clinical outcomes and a lower risk of arrhythmias, highlighting the utility of multimodal imaging for both monitoring and prognostication [66,67].

Looking ahead, emerging targeted therapies and biomarkers offer the potential for more precise, individualized management. Novel biologic agents such as TNF-α inhibitors and other immunomodulatory drugs are being explored for refractory or steroid-resistant disease, directly targeting inflammatory pathways central to granuloma formation. Simultaneously, biomarkers of response, including circulating cytokines (TNF-α, soluble IL-2 receptor), imaging metrics (FDG uptake, LGE), and emerging molecular markers, enable real-time monitoring of disease activity and therapeutic efficacy. Integrating these tools into clinical practice may allow early identification of responders, prediction of relapse, and optimization of therapy duration and intensity, advancing a personalized medicine approach in cardiac sarcoidosis [22,66]. Looking beyond conventional cardiologic devices, there is a rapidly growing interest in the application of advanced tissue engineering to address the electrical instability caused by fibrotic scarring. Specifically, the development of conductive biomaterials offers promising new avenues for the electrical pacing required to trigger cardiomyocyte contraction. Recent innovations, such as PEDOT:PSS-based microstructured electrodes and self-doped, biodegradable glycosaminoglycan-PEDOT conductive hydrogels, have demonstrated significant success in pacing induced pluripotent stem cell (iPSC)-derived cardiomyocytes within in vitro cell cultures. These highly biomimetic platforms not only provide exceptional in vitro models for studying the electrophysiological disruptions inherent to cardiac sarcoidosis, but they also firmly strengthen the need to invest resources in early prognosis and localized, bioengineered disease treatments [67,68].

## 11. Future Directions

Recent advances in the characterization of cardiac sarcoidosis (CS) have shifted focus toward novel biomarkers, artificial intelligence (AI), and quantitative imaging parameters that transcend traditional diagnostic criteria. A significant evolution is the integration of T2 mapping in cardiac magnetic resonance (CMR), which now allows for the precise quantification of myocardial edema, thereby distinguishing active inflammatory phases from established fibrosis with greater sensitivity than conventional Late Gadolinium Enhancement (LGE). Complementing this, AI-driven deep learning algorithms are increasingly utilized to automate the segmentation of FDG-PET and CMR datasets, enhancing inter-observer reproducibility and enabling the detection of sub-clinical imaging signatures that escape visual assessment. Parallel to imaging, the diagnostic landscape is expanding to include emerging biomarkers such as high-sensitivity cardiac troponin I (hs-cTnI) for disease activity monitoring and urinary 8-hydroxy-2′-deoxyguanosine (U-8-OHdG), a marker of oxidative DNA damage that has shown predictive value for ventricular arrhythmias. Furthermore, the identification of novel serum autoantibodies specifically anti-heart (AHA) and anti-intercalated disk (AIDA) antibodies suggests a potential autoimmune basis for the disease, offering new avenues for non-invasive risk stratification and targeted therapeutic intervention in serologically positive cohorts [22,69,70,71,72].

The elucidation of the specific molecular pathways driving cardiac sarcoidosis is opening new frontiers in precision therapeutics. By moving beyond broad immunosuppression, emerging strategies are focusing on targeting the specific intracellular drivers of granuloma formation. For instance, the central role of the JAK/STAT pathway in driving Th1-mediated inflammation has made JAK inhibitors (such as tofacitinib) a promising area of investigation for steroid-refractory cases. Similarly, addressing macrophage metabolic dysregulation through mTOR pathway inhibitors presents a highly specific molecular target to halt granuloma progression. Furthermore, the application of single-cell RNA sequencing (scRNA-seq) to endomyocardial biopsy specimens promises to unravel the molecular heterogeneity of the disease, allowing for the identification of patient-specific transcriptomic signatures that could guide individualized, molecularly targeted therapy [48,73].

## 12. Concluding Remarks

Sarcoidosis is a systemic granulomatous disorder of complex and multifactorial etiology, arising from the interplay of genetic susceptibility, environmental exposures, and immune dysregulation. While pulmonary involvement remains the most common manifestation, extrapulmonary sites, including the heart, kidneys, skin, eyes, and nervous system, can be affected, contributing significantly to morbidity and mortality. Cardiac sarcoidosis (CS), in particular, is frequently underdiagnosed due to its often subclinical presentation, yet it is a major determinant of adverse outcomes, including arrhythmias, conduction disturbances, and heart failure. Histopathologically, the hallmark non-caseating granulomas reflect a Th1-driven immune response, with progression to myocardial fibrosis associated with a shift toward Th2-dominant cytokine activity and worse prognosis.

Advances in non-invasive imaging, particularly cardiac magnetic resonance (CMR) and fluorodeoxyglucose positron emission tomography (FDG-PET), have revolutionized the detection, characterization, and monitoring of CS. These modalities, when integrated with targeted endomyocardial biopsy, provide a multimodal approach that enhances diagnostic sensitivity, facilitates precise localization of inflammatory lesions, and informs therapeutic decision-making. Speckle-tracking echocardiography and advanced imaging analysis further improve the ability to distinguish CS from phenocopies such as myocarditis, hypertrophic cardiomyopathy, or cardiac amyloidosis. The combined use of clinical assessment, imaging, histopathology, and molecular markers, including inflammatory cytokines and HLA-DR expression, enables a more precise evaluation of disease activity, prognosis, and risk stratification.

Management of CS relies on a multidisciplinary approach integrating immunosuppressive therapy and cardiologic interventions. Corticosteroids remain the first-line treatment for active inflammation, with adjunctive immunosuppressive agents such as methotrexate, azathioprine, and TNF-α inhibitors reserved for refractory or steroid-intolerant cases. Concurrent cardiologic care, including implantable cardioverter-defibrillators and guideline-directed heart failure therapy, addresses the significant risk of arrhythmias and ventricular dysfunction. Sequential imaging with PET and CMR allows clinicians to monitor therapeutic efficacy, guide immunosuppressive titration, and identify persistent inflammation or fibrotic progression, thereby enabling timely, personalized intervention.

Looking forward, emerging targeted therapies and predictive biomarkers hold promise for a precision medicine approach in sarcoidosis. The integration of molecular, imaging, and immunological markers may allow early identification of high-risk patients, more accurate prognostication, and individualized treatment strategies aimed at suppressing granulomatous inflammation while minimizing adverse effects. Continued research into the immunopathogenesis, imaging signatures, and molecular underpinnings of cardiac and systemic sarcoidosis is essential to improve patient outcomes, reduce disease burden, and establish standardized, evidence-based diagnostic and therapeutic guidelines.

## Figures and Tables

**Table 1:** Diagnostic Staining Patterns and Histopathological Findings in Cardiac Sarcoidosis.

Staining/ Modality	Target Component & Mechanism	Typical Cardiac Findings in Sarcoidosis	Diagnostic Utility & Interpretation	Advantages	Limitations
H&E (Hematoxylin & Eosin)	General tissue architecture; nuclei (blue/purple) and cytoplasm (pink).	Discrete, non-caseating epithelioid granulomas containing activated macrophages and multinucleated giant cells (Langhans type); often with a sparse lymphocytic rim.	Primary Diagnostic Method. Identifies the hallmark granuloma. Sensitivity is limited by the “patchy” distribution of lesions.	Widely available, rapid, and cost-effective; excellent for a broad morphological overview.	Low sensitivity due to the “patchy” distribution of lesions; cannot definitively rule out infectious mimics alone.
Masson’s Trichrome	Collagen fibers (stains blue) vs. cardiac muscle/cytoplasm (stains red).	Dense blue-stained fibrosis replacing red cardiomyocytes. Fibrosis is often extensive, surrounding granulomas or appearing as scar tissue.	Assessment of Chronicity. Distinguishes irreversible fibrotic scarring from active cellular inflammation; extent correlates with prognosis.	Clearly delineates the extent of structural remodeling and chronicity, which correlates with patient prognosis.	Does not identify the underlying etiology of the fibrosis; active inflammation may be obscured in dense scars.
IHC: CD68	Macrophage/Histiocyte lineage (lysosomal protein).	Strong positive staining in the granuloma core, highlighting epithelioid histiocytes and giant cells.	Confirmatory. Confirms the macrophage origin of the granuloma core, differentiating it from lymphocytic myocarditis	Highly sensitive for identifying macrophage aggregates, even in early or poorly formed granulomas.	Non-specific for sarcoidosis; also stains positive in infectious and other foreign-body granulomas.
IHC: CD3/CD4	T-Lymphocytes (CD3); T-Helper cells (CD4).	CD3+ lymphocytes form a peripheral cuff. CD4+ cells typically predominate over CD8+ cells.	Immunophenotyping. Reflects the Th1-mediated immune dysregulation characteristic of sarcoidosis	Elucidates the specific immune profile, aiding differentiation from predominantly cytotoxic (CD8+) viral myocarditis.	CD4/CD8 ratios can vary by disease stage; provides supportive rather than definitive diagnostic evidence.
Ziehl–Neelsen (ZN)	Acid-fast bacilli (mycolic acid in cell walls).	Negative. No acid-fast bacilli (red rods) detected.	Mandatory Exclusion. Rules out Mycobacterium tuberculosis (Tuberculous Myocarditis), a major mimic.	Essential for differentiating non-caseating sarcoidosis from tuberculosis; highly specific for acid-fast organisms.	Low sensitivity in low-burden mycobacterial infections; a negative stain ideally requires PCR confirmation.
PAS & GMS	Fungal cell walls, polysaccharides (PAS: magenta; GMS: black).	Negative for fungal hyphae or yeast forms within the granuloma.	Mandatory Exclusion. Rules out fungal myocarditis (e.g., Histoplasma, Aspergillus).	Excellent contrast for visualizing fungal structures against the myocardial background.	Non-contributory if the fungal burden is extremely low; only serves as a diagnosis of exclusion.
Electron Microscopy (EM)	Ultrastructural cellular details.	Complex interdigitation of epithelioid cells; Schaumann bodies or Asteroid bodies within giant cells	Adjunctive. Provides high-resolution confirmation of non-viral, non-bacterial etiology in equivocal cases.	Unmatched ultra-high-resolution detail; visualizes macrophage fusion and excludes viral particles/mycobacterial fragments.	Technically demanding, expensive, subject to severe sampling bias due to tiny tissue size, and prone to fixation artifacts.

## Data Availability

No new data were created or analyzed in this study. Data sharing is not applicable to this article.
